# Evaluating the Relationship between Spermatogenic Silencing of the X Chromosome and Evolution of the Y Chromosome in Chimpanzee and Human

**DOI:** 10.1371/journal.pone.0015598

**Published:** 2010-12-14

**Authors:** Eskeatnaf Mulugeta Achame, Willy M. Baarends, Joost Gribnau, J. Anton Grootegoed

**Affiliations:** Department of Reproduction and Development, Erasmus MC - University Medical Center, Rotterdam, The Netherlands; The Salk Institute, United States of America

## Abstract

Chimpanzees and humans are genetically very similar, with the striking exception of their Y chromosomes, which have diverged tremendously. The male-specific region (MSY), representing the greater part of the Y chromosome, is inherited from father to son in a clonal fashion, with natural selection acting on the MSY as a unit. Positive selection might involve the performance of the MSY in spermatogenesis. Chimpanzees have a highly polygamous mating behavior, so that sperm competition is thought to provide a strong selective force acting on the Y chromosome in the chimpanzee lineage. In consequence of evolution of the heterologous sex chromosomes in mammals, meiotic sex chromosome inactivation (MSCI) results in a transcriptionally silenced XY body in male meiotic prophase, and subsequently also in postmeiotic repression of the sex chromosomes in haploid spermatids. This has evolved to a situation where MSCI has become a prerequisite for spermatogenesis. Here, by analysis of microarray testicular expression data representing a small number of male chimpanzees and men, we obtained information indicating that meiotic and postmeiotic X chromosome silencing might be more effective in chimpanzee than in human spermatogenesis. From this, we suggest that the remarkable reorganization of the chimpanzee Y chromosome, compared to the human Y chromosome, might have an impact on its meiotic interactions with the X chromosome and thereby on X chromosome silencing in spermatogenesis. Further studies will be required to address comparative functional aspects of MSCI in chimpanzee, human, and other placental mammals.

## Introduction

In the evolution of the *Hominidae* family, humans shared a common ancestor with chimpanzees about 6 million years ago [1]. The human (*Homo sapiens*) and chimpanzee (*Pan troglodytes*) genomes are very similar, both in chromosomal organization and gene content [2]. However, the Y chromosomes of human and chimpanzee show remarkable differences [3], [4], [5]. The present mammalian X and Y sex chromosomes are the heterologous end products of an evolutionary cascade which started from a homologous pair of autosomes around 200 million years ago, before the separation of the marsupial and placental mammalian lineages [6], [7]. Next to small pseudo-autosomal regions (PARs), which are shared between X and Y, the greater part of the human Y chromosome consists of a male-specific region (MSY) that contains the male sex-determing gene *SRY*, and a total of 16 X-degenerate genes [5], [8] which contribute to gene dosage compensation between female XX cells and male XY cells [9]. In addition, the human MSY contains multicopy genes with ∼60 transcription units in ampliconic regions, encoding 9 different proteins mainly implicated in sperm production [5], [8]. Finally, the human Y has gained an X-transposed region with two genes, not present in the Y chromosomes of chimpanzee and other primates [5], [8]. When the chimpanzee Y chromosome was partially sequenced, by Hughes et al. in 2005, it was found that not only the X-transposed genes are missing in chimpanzee, but also 4 of the 16 X-degenerate genes appeared to be disrupted in the chimpanzee lineage [3].

The MSY is clonally inherited from father to son, and natural selection acts on the MSY as a unit. It is to be expected that the promiscuous mating behaviour of chimpanzees is associated with a high level of sperm competition, where the need for an increased quantity and quality of sperm production would be a driving force in selection [3], [10], [11]. Hence, it was suggested that gene loss from the X-degenerate region of Y might be compensated by a gain of function elsewhere on the chimpanzee Y chromosome, possibly regarding the ampliconic genes involved in spermatogenesis [3]. Surprisingly, with the complete sequencing of the chimpanzee Y chromosome by Hughes et al. in 2010, it was found that the ampliconic part of the chimpanzee Y chromosome also has lost genes, now containing only 25 genes encoding 7 different proteins [5]. The chimpanzee whole MSY region encodes 18 different proteins (37 genes), as opposed to the 27 different proteins (78 genes) that are encoded by the human MSY. This leaves us with the intriguing question how the chimpanzee Y chromosome has been optimized for sperm production. The ‘wholesale renovation’ of its structure [5] might affect transcription rates of individual genes. On the other hand, we anticipated that the interaction between the X and Y chromosomes needs to be taken into account.

In male meiotic prophase, the heterologous X and Y chromosomes pair and recombine only in the small PARs, and they form the transcriptionally silenced XY body [12], [13]. This inactivation is independent of *Xist*, and is referred to as meiotic sex chromosome inactivation (MSCI) [14], [15], [16]. Transcriptional silencing of the sex chromosomes, in particular that of the X, is partly maintained following completion of the meiotic divisions, when each haploid round spermatid contains either an X or a Y chromatid strand. This postmeiotic repression of the sex chromosomes is a downstream consequence of MSCI [17], [18]. MSCI itself can be considered as one of the consequences of the evolution of the heterologous X and Y chromosomes, which exposes these chromosomes to a conserved mechanism for meiotic silencing of unsynapsed chromatin (MSUC) [19], [20], [21]. This silencing is associated with a number of other evolutionary adaptations, including expression of a relative abundance of X-chromosomal genes in spermatogonia preceding meiotic prophase [22], [23], meiotic expression of a number of X-to-autosomal retrogenes replacing X chromosomal genes in spermatogenesis [24], [25], and, at least in the mouse, postmeiotic expression of X-linked multicopy genes that may escape from postmeiotic repression more readily than single-copy genes [26]. Together, this has evolved to a complex but balanced situation.

In mammalian species, meiotic X-Y interaction is a prerequisite for spermatogenesis [27], [28]. Related to this, MSCI also has become a prerequisite for spermatogenesis, so that deficiency of MSCI leads to dysregulation of spermatogenesis [16], [29]. From this, we hypothesize that a higher level of sperm competition, as found in chimpanzee as compared to human, might be associated with positive selection towards optimization of meiotic X-Y interaction and MSCI.

In a comparison of the number of expression changes between human and chimpanzee, for all individual chromosomes in five different tissues, a significant change in the expression of X-chromosomal genes has been detected [30]. Herein, we have investigated if this difference might be compatible with a differential level of MSCI, in particular X chromosome transcriptional silencing, in chimpanzee and human testes. The results are in agreement with our hypothesis that the chimpanzee X and Y chromosome pair may have gained the best performance in MSCI, compared to the human sex chromosomes, after we have separated from our closest relative.

## Results

### Expression of autosomal and X-chromosomal genes

An important resource for chimpanzee and human gene expression data, representing several organs from five male chimpanzees and six men, was reported by Khaitovich et al. (2005) [30]. The testis was shown to have the highest level of expression divergence between chimpanzee and human, compared to heart, liver, kidney and brain. Moreover, across all chromosomes in testis, most expression changes were detected for the X chromosome [30].

Using these valuable expression data, we have expanded the analysis. We averaged the expression of all individual probes in either brain, liver, or testis, for the five and six male individuals of the two species. Then, we selected all probes with a signal intensity of at least 100. A cut-off of 100 is generally applied in this type of analysis to eliminate noise from a low level of leaky expression. This is demonstrated for example by the analysis of global gene expression in human fetal gonads [31]. Steroidogenic genes are expressed in fetal testis at a level around 1000, whereas they are detected at a level around 100 in fetal ovary which is known to lack steroidogenesis. In a reciprocal manner, meiotic genes are expressed in fetal ovary at a level around 1000, whereas they are detected at a level around 100 in fetal testis which is known to lack meiosis [31]. For the present analysis, we next calculated the average expression level for all autosomal probes (AEL) and the average expression level for all X chromosomal probes (XEL), for chimpanzee and human brain, liver, and testis. The first two organs were selected for the present analysis as the non-gonadal organs with respectively the highest and lowest level of gene expression diversity between individuals in each of the two species [30].

**Figure 1:**
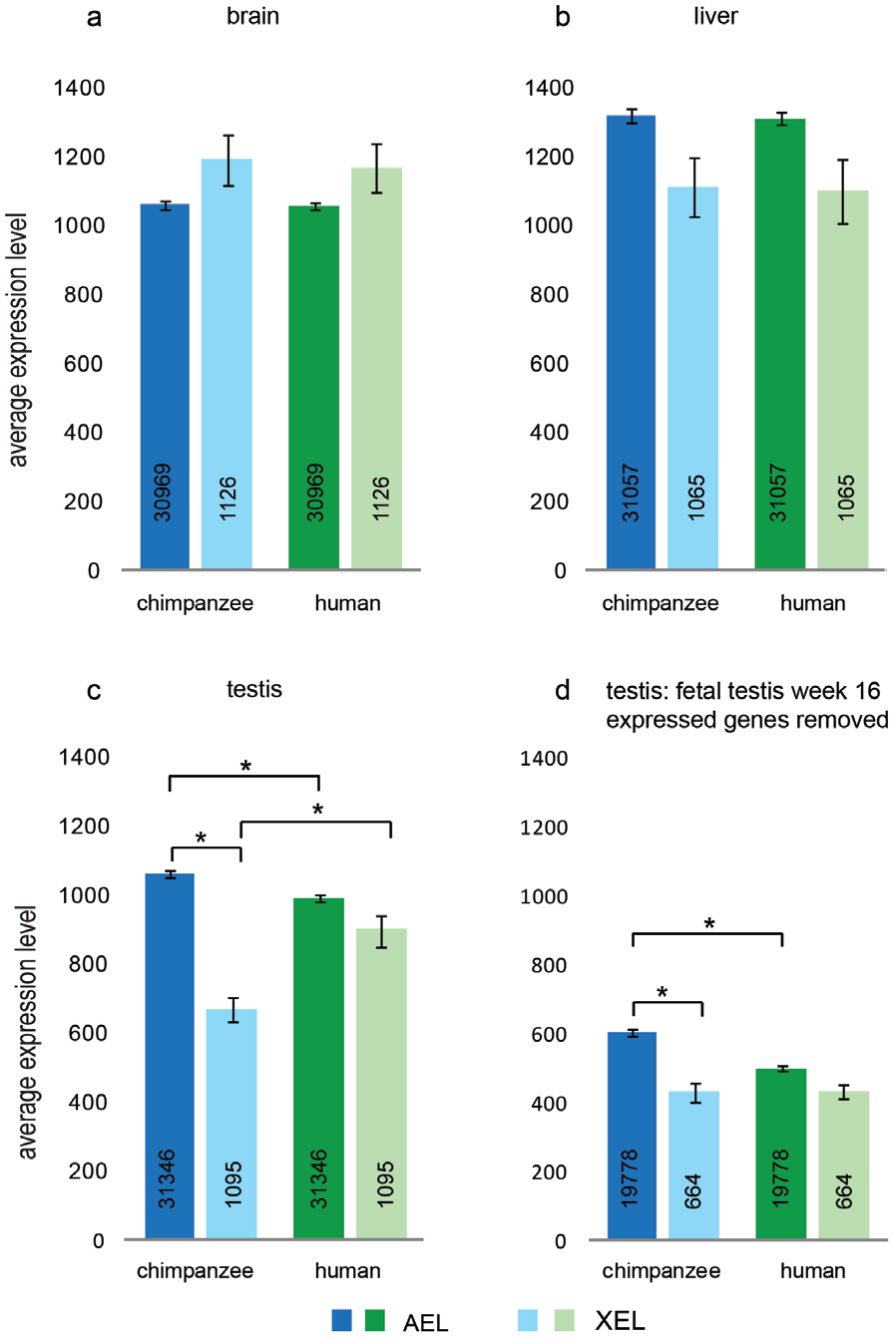
Average expression level of probes linked to all autosomes or the X chromosome. Probes were annotated with their chromosomal location and the average expression level was calculated for autosomal genes (AEL) and X-chromosomal genes (XEL) in chimpanzee and human brain (a), liver (b), and testis (c). Panel (d) shows the AEL and XEL for testis, after removal of all probes representing genes expressed in human week 16 fetal testis. Error bars represent SEM, and an asterisk (*) indicates a statistical significance (Wilcoxon rank sum test) between groups. In c): AEL in human testis lower than AEL in chimpanzee testis (p-value 2.2e-16); XEL in chimpanzee testis lower than AEL in chimpanzee testis (p-value 1.1e-10); XEL in chimpanzee testis lower than XEL in human testis (p-value 8.8e-08). In d): AEL in human testis lower than AEL in chimpanzee testis, after removal of human week 16, fetal testis expressed genes (p-value 5.0e-05); XEL in chimpanzee testis lower than AEL in chimpanzee testis, after removal of human week, 16 fetal testis expressed genes (p-value 4.7e-05).

In addition to gene dosage compensation between male and female cells by X chromosome inactivation in female cells [32], [33], the hemizygous condition of males regarding the X chromosome is compensated by a two-fold transcriptional upregulation of the mammalian X chromosome in most somatic cell types [9], [34], [35]. This explains why male brain and liver have an XEL which is not two-fold lower than that of the AEL, but rather is within the same range (Figure 1a,b). For AEL and XEL in brain and liver, no significant difference was observed between chimpanzee and human (Figure 1a,b).

For chimpanzee testis, the XEL was found to be much lower than the AEL (Figure 1c). This would be in agreement with MSCI impacting on global X-chromosomal gene expression. Much of the testis tissue is comprised of the germ cell types in which MSCI and postmeiotic repression take place, and it is to be expected that X chromosome silencing during spermatogenesis leads to a lower XEL, as compared to the AEL, when expression data are analyzed for the whole tissue. However, the human testis showed an XEL not significantly lower than the AEL (Figure 1c). This might reflect that MSCI is less effective in human testis, provided that differences in cellular composition of the chimpanzee and human testes do not mask most of the effect of MSCI on XEL in human. Several reports indicate that differences in sperm output among primate species are not associated with marked differences in relative numbers of germ cell types [30], [36]. For human testis, 1.13×10^9^/testis pachytene spermatocytes and 3.95×10^9^/testis round/elongating spermatids per 8.29×10^9^/testis total germ cells and Sertoli cells have been counted, using a stereological technique, the optical disector [37]. This implies that the percentage of spermatocytes and round/elongating spermatids is approximately 14% and 48% of all cells in the spermatogenic epithelium, respectively. Using flow cytometry of whole testis from 12 primate species (prosimians, and new world and old world monkeys), 10–15% primary spermatocytes and 40–55% round spermatids have been counted [36], [38]. A direct comparison with the chimpanzee has not been reported, but it appears that the cellular composition of human testis tubules at least is very similar to that of many other primates. However, this may not apply to the amount of interstitial cells. We do not exclude that the relative amount of interstitial tissue of chimpanzee and human testes are quite different. Hence, we performed an additional analysis, by using microarray data representing human fetal testis at 16 weeks of gestation [31], when the testis contains a fetal population of Leydig cells active in steroidogenesis, in addition to peritubular cells, immature Sertoli cells, and spermatogonial stem cells. We selected all probes expressed at 16 weeks of fetal development (cut-off at 100) and removed these probes from the analysis of the adult chimpanzee and human testes. This will result in a loss from the analysis of many autosomal and X-linked genes expressed in somatic testicular cell types or spermatogonial stem cells, and also in a loss of autosomal and X-linked house-keeping genes. Concomitantly, we expect an enrichment of autosomal and X-linked genes specifically expressed in spermatocytes and spermatids, not present in the fetal gonads. The result of this selection is shown in Figure 1d. The AEL was found to be decreased compared to Figure 1c, reflecting that high-expressed house-keeping genes are not represented and that the analysis is focused on a smaller percentage of testicular cells. The AEL in human testis was approximately 20% lower than that in chimpanzee, which indicates that per unit of tissue the human testis contains around 20% less germ cells than the chimpanzee testis. The relatively low XEL in chimpanzee testis, compared to the AEL, was observed in both analyses presented in Figure 1c and 1d, indicating that a decreased XEL occurs mainly if not exclusively in the germ cells. This reinforces that MSCI and post-meiotic repression of X-linked genes might be more effective in chimpanzee testis, compared to human testis.

It cannot be excluded that low-expressed genes respond differently to MSCI as compared to high-expressed genes. This information is lost by global analysis and arithmetic averaging of expression levels. We re-analysed Figure 1c without applying the cut-off of 100, but this did not markedly change the pattern observed (Figure S1).

Mutation rates vary among chromosomes [39] and the human-chimpanzee genetic divergence shows variation between autosomes [40]. Therefore, we also analyzed the average expression level of chromosomes 3, 6 and 8, which have a length and gene density comparable to that of the X chromosome; chromosomes 6 and 8 show a relatively high chimpanzee-human divergence [40]. The results of this analysis showed the absence of a marked difference in the average expression level (3EL, 6EL, and 8EL) of the selected autosomes 3, 6 and 8 (Figure S2). This result confirms that the X chromosome shows a relatively low average expression level compared to autosomes, also when compared to individual autosomes, in chimpanzee testis.

In a different type of analysis of the available data, we established the *numbers* of differentially expressed genes between chimpanzee and human testes. We found that a larger percentage of autosomal genes (46%) compared to a smaller percentage of X-linked genes (28%) is expressed at a higher level in chimpanzee testis (Figure S3). In a reciprocal fashion, a smaller percentage of autosomal genes (32%) compared to a larger percentage of X-linked genes (42%) was found to be expressed at a lower level in chimpanzee testis (Figure S3). The result of this analysis also is in agreement with the suggestion that MSCI and postmeiotic repression might be more effective in chimpanzee testis, compared to human testis.

### Meiotic and postmeiotic changes in gene expression

The above expression data concern whole testis tissue, whereas we would need to obtain information about specific steps in spermatogenesis. It would not be feasible to isolate germ cell types from chimpanzee and human testis, from several males, under the same experimental conditions, and resulting in comparable purity and viability of the cell populations. Therefore, we used microarray gene expression data obtained for isolated germ cell types from mouse testis by Namekawa et al. (2006) [18] in the next step of our study. In view of conserved cellular aspects of spermatogenesis, we anticipate that most genes will show conservation of their expression patterns at subsequent steps of spermatogenesis, between mouse and primates, and we used the available mouse microarray data to gain information about spermatogenic gene expression patterns in whole chimpanzee and human testes.

**Figure 2:**
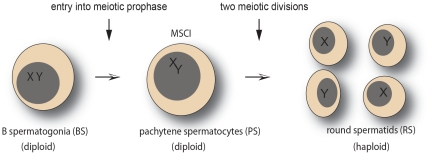
Schematic presentation of two developmental steps in spermatogenesis. The meiotic silencing of sex chromosomes (MSCI) is included in the step from B spermatogonia (BS) to pachytene spermatocytes (PS). The step from PS to round spermatids (RS) includes the transition from MSCI to postmeiotic repression.

In the last mitotic division of spermatogenesis, B spermatogonia give rise to primary spermatocytes entering into meiotic prophase. At the end of the lengthy meiotic prophase, which includes the pachytene stage, the first meiotic division results in the formation of haploid secondary spermatocytes. The second meiotic division gives rise to the haploid round spermatids. This is followed by extensive cytodifferentiation of spermatids, supported by the expression of testis-specific genes, to become elongated and condensed spermatozoa (reviewed in [41]). Isolation of mouse germ cell types is a well established approach, used to obtain microarray gene expression data for specific germ cell types [18]. For the present study, we selected as the most relevant cell types: B spermatogonia (BS; premeiotic cells), pachytene spermatocytes (PS; primary spermatocytes in meiotic prophase), and round spermatids (RS; postmeiotic haploid cells). In the analysis, we focused on the step from BS to PS (which includes MSCI in primary spermatocytes), and the step from PS to RS (which includes the transition from MSCI to postmeiotic repression) (Figure 2). For both steps, we selected all probes that were either upregulated or downregulated (as described in Methods).

In mouse and primates, the X chromosome carries about 1000 genes of the haploid genome of around 23000 genes, so that in a diploid XY male the total number of different autosomal genes is about 22-fold the number of X chromosomal genes. Hence, the 22X:A figures presented in Table 1 provide an estimate of the ratio between expressed X chromosomal genes and expressed autosomal genes. For all probes up- or downregulated in the two steps BS to PS and PS to RS taken together, the calculated 22X:A was found to equal 1.0. For genes that are downregulated in the step BS to PS in mouse, we observed a 22X:A figure of 1.9 (Table 1). This is consistent with silencing of the X chromosome by MSCI. Relatively small numbers of X chromosomal probes represent genes that are either upregulated in the step BS to PS (22X:A of 0.3) or downregulated in the step PS to RS (22X:A of 0.2) (Table 1). This is in agreement with MSCI as a dominant factor in the downregulation of the X chromosome, in the step BS to PS, which also implies that few X chromosomal genes will be candidate for further downregulation postmeiotically, in the step PS to RS. A relatively large number of X chromosomal probes (22X:A of 2.0) showed upregulation in the step PS to RS (Table 1), and this will include genes escaping partly or completely from postmeiotic repression following MSCI, as well as genes that are first expressed in round spermatids.

**Table 1:** Matching of probes expressed in chimpanzee and human testes to probes differentially expressed at subsequent steps of spermatogenesis in mouse.

	BS to PS *down*	matched probes	BS to PS *up*	matched probes
	mouse	chimp/human	mouse	chimp/human
autosomal	5997	4538	5603	3537
X-linked	516	366	57	25
22X:A	1.9	1.8	0.2	0.2

	PS to RS *down*	matched probes	PS to RS *up*	matched probes
autosomal	2545	1750	2212	1312
X-linked	33	20	200	96
22X:A	0.3	0.3	2.0	1.6

The mouse probes represent genes down- or upregulated in the step from BS (B spermatogonia) to PS (pachytene spermatocytes), or down- or upregulated in the step from PS to RS (round spermatids) [18]. Matching of the chimpanzee and human probes to the mouse probes was done based on gene symbols, as described in Methods. Few probes may have escaped from this matching or may have been matched to different genes for the different species, but we expect that this has no significant impact on the current global analysis. New X-linked genes, mainly duplications, arising after the chimpanzee-human split [45] are not included in the present analysis, which is based on a human microarray, and the numbers of matched probes associated with the four different developmental expression patterns were identical for chimpanzee and human.

**Figure 3:**
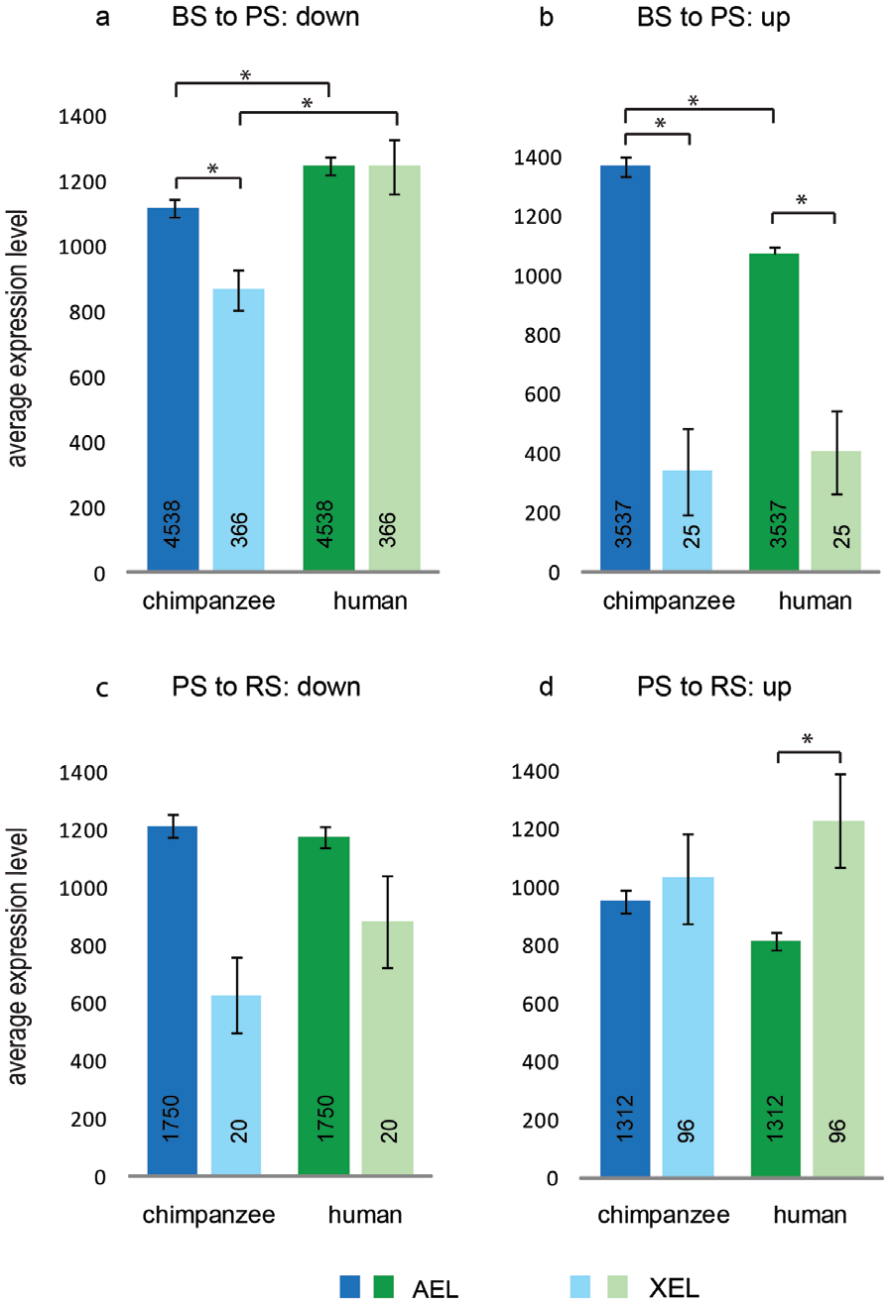
Differential gene expression at subsequent developmental steps of spermatogenesis. Chimpanzee and human autosomal and X-linked probes expressed in testis were mapped to mouse probes that show differential expression at subsequent steps of mouse spermatogenesis. The average expression level for autosomal genes (AEL) and that for the X-chromosomal genes (XEL) represent the expression in chimpanzee and human testes for probes that are: a) downregulated in the BS-PS step; b) upregulated in the BS-PS step; c) downregulated in the PS-RS step; d) upregulated in the PS-RS step. Error bars represent SEM, and an asterisk (*) indicates a statistical significance (Wilcoxon rank sum test) between groups. In a): AEL in chimpanzee lower than AEL in human (p-values 2.2e-16); XEL in chimpanzee lower than XEL in human (p-value 5.9e-7); XEL in chimpanzee lower than AEL in chimpanzee (p-value 3.8e-3). In b): AEL in chimpanzee higher than AEL in human (p-value 9.3e-3); XEL in chimpanzee lower than AEL (p-value 4.4e-7); XEL in human lower than AEL (p-value 2.0e-5). In d): XEL in human higher than AEL (p-value 1.6e-3).

The chimpanzee and human whole testis probes were matched to the mouse probes representing the four different expression patterns (either down or up in the step BS to PS, and either down or up in the step PS to RS), based on gene symbols, as described in Methods. The matched probes showed 22X:A ratios comparable to the ratios obtained for the mouse probes (Table 1). Next, the AEL and XEL for the matched probes expressed in chimpanzee and human testes were calculated (Figure 3).

In the step BS to PS, significant differences were observed between chimpanzee and human for the AEL of down- or up-regulated genes (Figure 3a, 3b). This is in agreement with the results shown in Figure 1, and may represent a lower percentage of tubular cells compared to interstitial cells, in human testis. Similar to Figure 1d, the analysis presented in Figure 3 is focused on genes expressed in spermatogenic cells, given the fact that we select genes that are down- or upregulated in spermatogenic cells progressing through spermatogenesis.

For X-chromosomal genes downregulated in the step BS to PS, the XEL was much lower in chimpanzee than in human testis (Figure 3a). A relatively low XEL, compared to the AEL, is expected because MSCI occurs in pachytene spermatocytes (PS). For human testis, we found an XEL similar to the AEL also for the analysis of this selected group of genes (Figure 3a). In agreement with the results presented in Figure 1, this suggests that there might be a difference in the efficiency of MSCI in chimpanzee and human testes.

Few X chromosomal genes were found to be upregulated in the step BS to PS in mouse, and for the 25 matched *Hominidae* probes a very low XEL was calculated (Figure 3b). This is consistent with MSCI counteracting upregulation of X chromosomal genes in the step BS to PS, both in chimpanzee and human testes.

Similarly, only 20 matched probes in chimpanzee and human testis represented X-chromosomal genes downregulated in the step PS to RS in mouse, and no statistical significant differences were observed (Figure 3c). A low number of probes representing genes downregulated in the step PS to RS is to be expected, because MSCI is most likely not followed by an even more complete silencing of the X chromosome, postmeiotically. Rather, postmeiotic repression will maintain the silencing at the same level, or genes will be released from silencing. Indeed, a higher number of X chromosomal genes was found to be upregulated in the step PS to RS (96 matched probes), and for these *Hominidae* probes we found a relatively high XEL, that was even higher than the AEL in human testis (Figure 3d). This points to an increased rate of escape from postmeiotic repression, in the human testis compared to the chimpanzee testis.

## Discussion

Meiotic silencing of sex chromosomes in spermatocytes (MSCI), resulting in the silenced XY body or sex body, does not require *Xist* RNA [14], [15], meaning that it occurs independent of the molecular machinery that carries out X chromosome inactivation in female mammalian somatic cells [32], [33]. Rather, the formation of the XY body is based on a highly conserved mechanism which silences all regions of unpaired chromatin in meiotic prophase, named meiotic silencing of unsynapsed chromatin (MSUC) [19], [20], [21], which is not male-limited and probably predates the evolution of the heterologous XY pair in mammals [29]. It seems very unlikely that such a conserved mechanism would be subject to rapid positive selection, in the chimpanzee lineage. Yet, the present results indicate that MSCI and the subsequent postmeiotic repression of the X and Y chromosomes is pronounced in chimpanzee spermatogenesis, but less effective in human spermatogenesis. Multifarious factors or processes might effectuate a relatively high effectiveness of X chromosome silencing in chimpanzee spermatogenesis.

The X chromosome is not resistant to evolutionary adaptation. As predicted by Susumu Ohno (1967) [42], mammalian species have conserved the gene content of the X chromosome, which is explained by constraints related to dosage compensation of X-linked genes between females and males. This is known as Ohno's law, which holds true in general, but there are exceptions such as a reported single gene contravention of Ohno's law in mice [43], changes with regard to gene retrotransposition on and off the X chromosome, and pronounced gene duplication events [22], [23], [26], [44], [45].

The hypothesis of “male-driven evolution” involves that male germline cells experience more mutational pressure than female germline cells, mainly related to a higher number of mitotic divisions required to generate spermatozoa throughout adult reproductive life [46], [47], [48], [49]. In placental mammals, the X chromosome spends only one-third of its time in males and hence is exposed to male-driven evolution to a lesser extent, compared to the autosomes. Indeed, synonymous substitutions between chimpanzee and human are less frequent on the X chromosome than on the autosomes [50]. However, from the analysis of coding sequences of large numbers of X-linked and autosomal genes, it also appeared that the rate of *non*synonymous substitutions between chimpanzee and human is higher on the X chromosome than on the autosomes [50]. X-linked genes are hemizygous in males, which advances the fixation of mutations associated with positive natural and sexual selection [51]. For X-chromosomal genes expressed in testis, Khaitovich et al. (2005) suggested positive selection of sequence changes as well as expression changes, leading to the X chromosome showing the highest number of expression changes between human and chimpanzee across all chromosomes in testis [30]. In the present differential expression analysis for all individual probes in chimpanzee versus human testes, we found that 28% and 42% of X-linked probes were expressed at a higher and lower level, respectively, in chimpanzee testis compared to human testis. This implies a remarkable shift towards lower expression of a large number of X-linked genes in chimpanzee testis, and it can be questioned what is the biological rationale behind positive darwinian selection of sequence changes acting towards such a lower expression. Rather, we suggest that a more global shift has occurred, independent of selection of sequence changes.

To try to explain such a global shift towards a differential average expression level of X chromosomal genes between chimpanzee and human testes, we suggest that interaction between the X and Y chromosomes needs to be taken into account. Evolutionary changes of the Y chromosome might impact on silencing of the X chromosome in spermatogenesis. Deletions on the mouse Y chromosome long arm have been shown to lead to dysregulation of spermatogenesis, associated with upregulation of transcripts from the X and Y chromosomes in spermatids [52]. Within the deleted mouse Yq region, the multicopy *Sly* gene (*Sycp3-like Y-linked*) was shown to encode a postmeiotic repressor of the sex chromosomes, and the encoded SLY protein colocalizes with X and Y chromatin in mouse spermatids [53]. These mouse studies indicate that dysregulation of postmeiotic repression can lead to male subfertility or infertility. A gene homologous to mouse *Sly* has not been found on the chimpanzee and human Y chromosomes, which however does not exclude that a similar mechanism, for Y to exert some control over postmeiotic repression, might be operative in other mammalian species including the *Hominidae*. Moreover, the Y chromosome can be expected to exert control over MSCI, in all mammalian species.

In female heterogametic birds, the W chromosome resembles the Y of mammals as being the degenerated member of the ZW sex chromosome pair. The heterologous Z and W engage in pairing in the meiotic prophase of oocytes, and we have demonstrated that the meiotic ZW pair in chicken oocytes undergoes transient silencing partly resembling mammalian MSCI [54], [55]. The W chromosome enters meiotic prophase carrying chromatin inactivation marks, and seems to engage the much larger Z chromosome in heterologous synapsis and silencing [54]. Engagement between the heterologous X and Y chromosomes in mammalian male meiotic prophase probably involves different mechanisms than the ZW interplay in avian female meiosis, but there might be intriguing similarities. For mouse, it has been shown that X and Y chromosomes display extensive side-by-side pairing early in meiotic prophase, which decreases in length as meiotic prophase progresses [56]. Similarly, pairing between the human X and Y chromosomes was found to be very extensive at early pachytene, where it could involve the entire MSY region of the Y chromosome [27]. When an X chromosome lacks a Y chromosome as pairing partner in meiotic prophase, this single X chromosome will be subject to silencing by the MSUC (meiotic silencing of unsynapsed chromatin) mechanism, but this is less effective. The single X can escape from MSUC by full non-homologous self-synapsis, forming a hairpin structure [16], [29], [57]. This escape from silencing of a single X chromosome in meiotic prophase also points to a role of the Y chromosome in MSCI of the paired X and Y chromosomes.

From the above, it is clear that we should take into account that the meiotic interactions of X and Y involve more than just pairing and synapsis of the pseudoautosomal regions. In mammalian sex chromosome evolution, the X chromosome has been adapted to the consequences of Y chromosome evolution, such as by accumulating changes in genetic content by positive natural and sexual selection, gaining a two-fold upregulation of its overall transcriptional activity, and undergoing inactivation of one of the two X chromosomes in female cells [32], [44], [58]. Simultaneously, the X and Y chromosomes have developed as a heterologous pair which has maintained pseudoautosomal regions important for pairing in meiotic prophase, and they may have gained an overall structure which allows for timely and efficient XY body formation in spermatogenesis. The human X chromosome and the chicken Z chromosome both show a relatively low gene density, compared to autosomes, resulting from an expansion of intergenic regions [59]. These two sex chromosomes have in common that they are shared between the sexes, in contrast to the Y and W sex chromosomes which are found only in XY men or in the ZW female chicken, respectively. Z chromosome dosage compensation in ZZ male birds is not very pronounced [60], [61], compared to the highly efficient X chromosome dosage compensation by X chromosome inactivation in XX female cells [32], [33]. However, MSCI of ZW in chicken oocytes appears to be quite effective [54], [55]. Possibly, some aspect of the convergent evolution of the chicken Z and the human X, such as the expansion of intergenic regions [59], might concern their meiotic interactions with W and Y, respectively, including their performance in MSCI. Different types of repeats and their associated chromatin modifications and chromatin-based events could be involved in a variety of meiotic interactions between the X and Y chromosomes. The chimpanzee Y chromosome contains twice as many massive palindromes as the human Y chromosome and these palindromes are distributed over the chimpanzee MSY in a comparatively regular pattern [5]. We suggest that this might provide the chimpanzee Y chromosome with a global architecture which promotes its meiotic interactions with the X chromosome in the pathway leading to MSCI.

In human, microdeletions of the Y chromosome leading to male subfertility or infertility concern the overlapping AZF (azoospermia factor) regions AZFa, AZFb, and AZFc (reviewed in [62]). Evidently, the hunt is on, to try to identify genes within these regions which are required for spermatogenesis. The gene *USP9Y*, located in the AZFa region, was considered a candidate male fertility gene, following the discovery of an inactivating mutation in a man with nonobstructive azoospermia, which was absent in his fertile brother [63]. However, natural transmission of *USP9Y* gene mutations has been observed [64], and the *USP9Y* gene appears to be missing from the chimpanzee Y chromosome [5]. Consequently, the putative indispensible role for *USP9Y* in sperm production is questioned. In fact, at present there is no gene known within the AZF regions which can explain any of the male infertility phenotypes associated with AZF deletions [65], [66]. Hence, there is room to suggest that AZF deletions might affect Y chromosome structure, rather than its critical gene content, to such an extent that MSCI is dysregulated, consequently leading to impairment of spermatogenesis. Speed and Chandley (1990) [67] reported on morphological abnormalities of the XY pair in spermatocytes of infertile men. An effect of Yq deletions on the X-Y pairing process, by changing the dynamic condensation behavior of the entire Y chromosome structure, was suggested by Vogt et al. (1995) [68]. A more recent report describes that AZFb-c deletions disturb X and Y chromosome pairing in meiotic prophase [69]. At the structural level, based on confocal microscopic analysis, the 3D parameters of the heterochromatic XY body in chimpanzee and human testis were found to be very similar [70], but it would be of much interest to study in more detail XY body formation and other aspects of X and Y structural and functional chromatin dynamics in chimpanzee and human spermatogenesis, including men carrying AZF deletions.

Within the *Hominidae* family, the loss of 4 of the 16 X-degenerate genes from the Y chromosome in the chimpanzee lineage has not occurred in human, and also not in *Gorilla gorilla* [71], [72]. Regarding mating behavior, the highly polygamous chimpanzee is contrasted by the pronounced monogamous gorilla, meaning that sperm competition is least relevant in gorilla, probably even less than in the human lineage. The sequence of the gorilla Y chromosome is still awaited for, so there is no complete information regarding loss or gain of other genes from the gorilla MSY. The MSY of human and gorilla might turn out to be quite similar, being subject mainly to negative selection [73], in contrast to positive selection driven by sperm competition reshaping the MSY in the chimpanzee lineage. This does not exclude that there might be some gain for human and gorilla for having kept genes in the MSY intact, which could concern functions of the Y chromosome in somatic cells and tissues, rather than in spermatogenesis.

To what extent a limited failure of meiotic sex chromosome inactivation (MSCI), including its downstream impact on postmeiotic gene expression in spermatogenesis, is tolerated in different mammalian species, is unknown. Further studies will be required to address comparative functional aspects of MSCI in chimpanzee, human, and other placental mammals. Yet, we suggest that evolutionary changes in the structure of the Y chromosome in the chimpanzee lineage might have an impact on its interactions with the X chromosome in male meiotic prophase, thereby affecting chromatin structure and transcriptional dynamics of the X chromosome in spermatogenesis, leading to a quantitative and qualitative improvement of sperm production.

## Methods

The microarray expression data for different tissues from five male chimpanzees and six men, generated using Affymetrix chip U133 plus 2 [30], were obtained from ArrayExpress repository (http://www.ebi.ac.uk/microarray-as/ae/), using accession number E-AFMX-11. For mouse testis and isolated germ cell types, microarray expression data, generated using the Affymetrix chip mouse genome 430 2.0 [18], were obtained from NCBI GEO (http://www.ncbi.nlm.nih.gov/geo/) using GEO accession number GSE4193. Microarray data representing human week 16 fetal testis [31]were obtained from NCBI GEO using GEO accession number GSE15431.

Data was normalised using RMA (robust multichip average) normalisation. Genes differentially expressed between chimpanzee and human testis, and genes that are differentially expressed at different stages of mouse spermatogenesis, were identified using Limma (linear models for microarray data) implemented in R software [74]. To calculate the average expression level of probes representing autosomal and X-linked genes in different chimpanzee and human tissues, probe id's were annotated with chromosome number and gene name (Affymetrix HG-U133 Plus2 Annotations, release 30). RMA normalised expression level was averaged among the 5 and 6 individuals of each species, for each probe, and low expressing probes with a signal intensity less than 100 in both species were excluded. The resulting data was used to calculate the average expression level and to perform statistical tests.

To calculate the percentage of differentially expressed (higher and lower; or no change) genes between chimpanzee and human testis, all probes were mapped to their respective genes and chromosomal locations. Duplicate probes that mapped to the same gene were averaged.

To investigate the level of expression of chimpanzee and human genes at different steps of spermatogenesis, we used the microarray data generated from RNA extracted from isolated mouse spermatogenic cell types [18]. Mouse probe id's that are differentially expressed at these different steps were annotated with mouse gene names (Affymetrix mouse genome 430 2.0 Annotations, release 20). Mouse genes names were then mapped to chimpanzee and human array gene names (Affymetrix HG-U133 Plus2 Annotations, release 30). The average expression level of probes in chimpanzee and human was calculated using data obtained from normalised chimpanzee and testis microarray data described above.

Statistical significance was calculated using R software. The error bars in the Figures represent SEM. An asterisk indicates when a statistical significance was detected, and the respective p-values are given in the Figure Legends.

## Supporting Information

Figure S1 Re-analysis of the AEL and XEL in chimpanzee and human testes as shown in Figure 1c, without using a cut-off of 100.
(TIF)

Figure S2 Average expression level in testis of probes linked to different chromosomes.
Probes were annotated with their chromosomal location and the average expression level in chimpanzee and human testes was calculated for all autosomes (AEL) and chromosomes 3, 6, and 8 (3EL, 6EL, and 8EL), as well as for the X chromosome (XEL).
(TIF)

Figure S3 Differential gene expression between chimpanzee and human testis.
Differentially expressed genes between chimpanzee and human were identified, mapped to their chromosomal location, and the percentage of genes with either higher or lower expression in the chimpanzee compared to the human testis is presented.
(TIF)
