# Supplementary figures and images for: Evaluating the Relationship between Spermatogenic Silencing of the X Chromosome and Evolution of the Y Chromosome in Chimpanzee and Human
Source: PLoS One. 2010 Dec 14;5(12):e15598. doi: 10.1371/journal.pone.0015598 (PMC3001880; doi:10.1371/journal.pone.0015598)

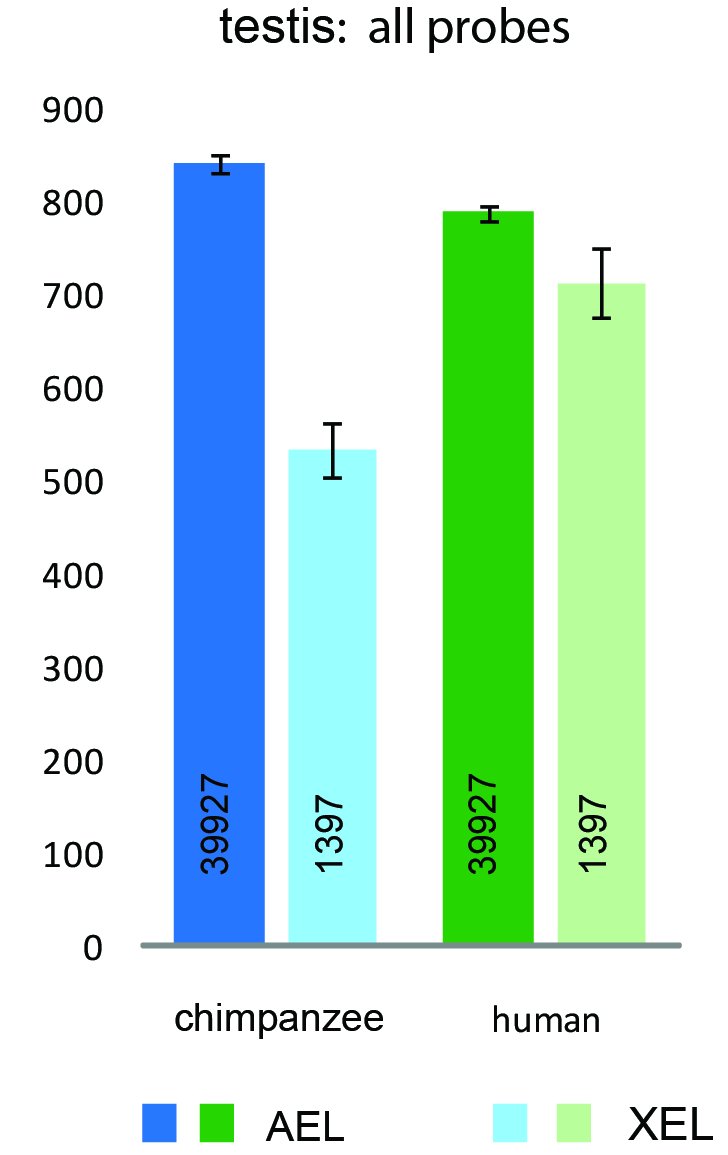

Supplement: Figure S1 — Re-analysis of the AEL and XEL in chimpanzee and human testes as shown in Figure 1c, without using a cut-off of 100. (TIF) [file pone.0015598.s001.tif]

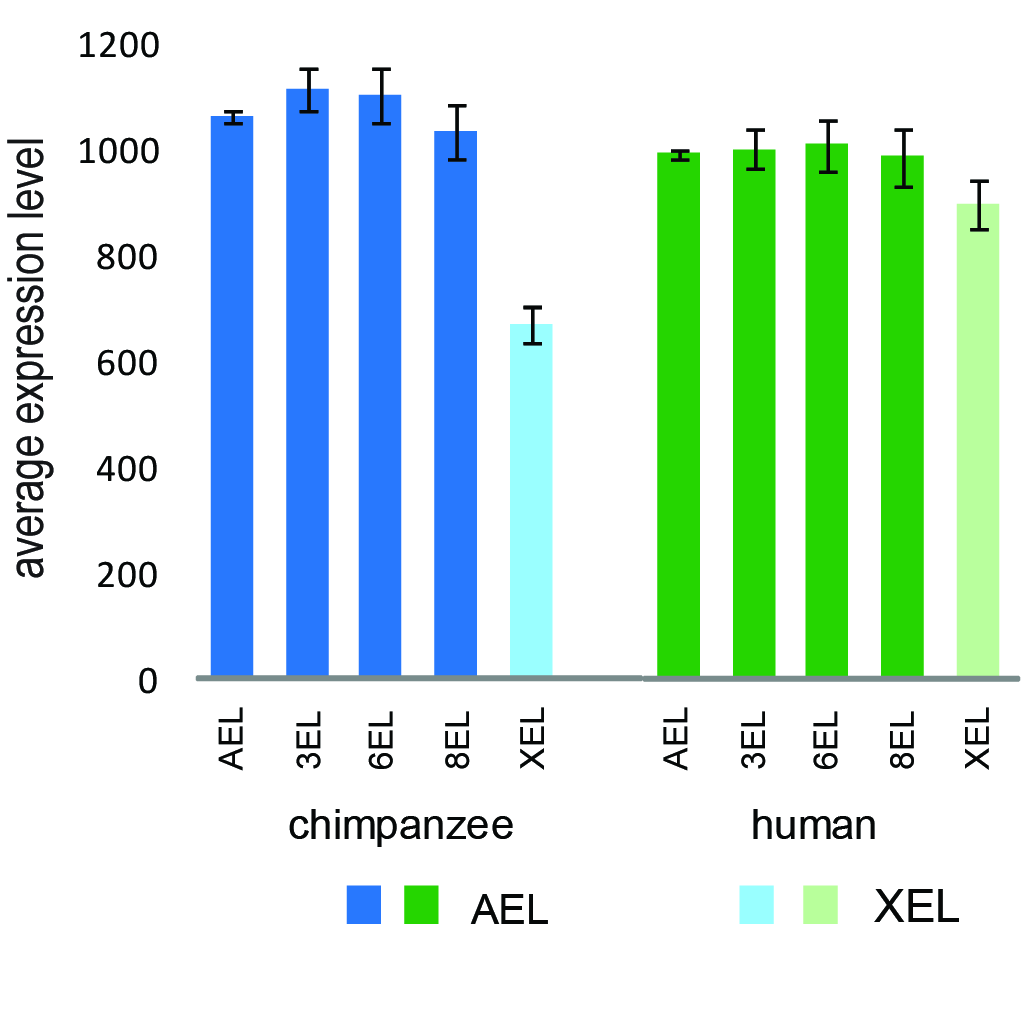

Supplement: Figure S2 — Average expression level in testis of probes linked to different chromosomes. Probes were annotated with their chromosomal location and the average expression level in chimpanzee and human testes was calculated for all autosomes (AEL) and chromosomes 3, 6, and 8 (3EL, 6EL, and 8EL), as well as for the X chromosome (XEL). (TIF) [file pone.0015598.s002.tif]

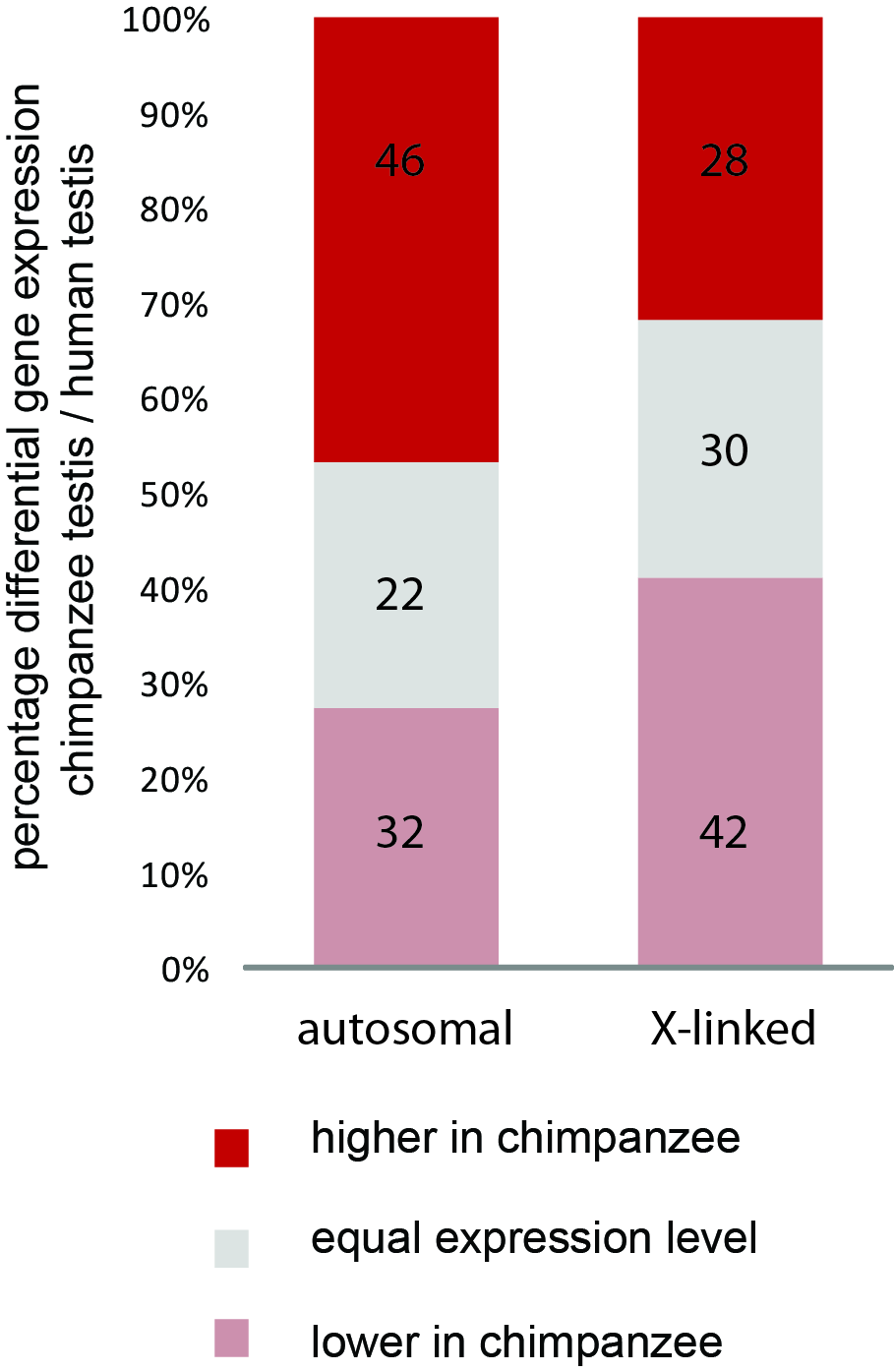

Supplement: Figure S3 — Differential gene expression between chimpanzee and human testis. Differentially expressed genes between chimpanzee and human were identified, mapped to their chromosomal location, and the percentage of genes with either higher or lower expression in the chimpanzee compared to the human testis is presented. (TIF) [file pone.0015598.s003.tif]
